# Supplementary material for: Effect of Cellulose Nanofibrils and TEMPO-mediated Oxidized Cellulose Nanofibrils on the Physical and Mechanical Properties of Poly(vinylidene fluoride)/Cellulose Nanofibril Composites
Source: Polymers (Basel). 2019 Jun 27;11(7):1091. doi: 10.3390/polym11071091 (PMC6680576; doi:10.3390/polym11071091)
Supplement: Supplementary file 1 [file polymers-11-01091-s001.pdf]

Effect of Cellulose Nanofibrils and TEMPO-mediated Oxidized Cellulose Nanofibrils on the Physical and Mechanical Properties of Poly(vinylidene fluoride)/Cellulose Nanofibril Composites

Supporting Information

Eftihia Barnes [1,*], Jennifer A. Jefcoat [1], Erik M. Alberts [2], Mason A. McKechnie [1], Hannah R. Peel [3], J. Paige Buchanan [1], Charles A. Weiss Jr. [1], Kyle L. Klaus [1], L. Christopher Mimun [3], Christopher M. Warner [3]

[1]Geotechnical and Structures Laboratory, U.S. Army Engineer Research and Development Center

3909 Halls Ferry Road, Vicksburg, Mississippi, USA

[2]HX5, LLC, Vicksburg, Mississippi, USA

[3]Environmental Laboratory, U.S. Army Engineer Research and Development Center

3909 Halls Ferry Road, Vicksburg, Mississippi, USA

*Correspondence: Eftihia.V.Barnes@usace.army.mil; Tel.: +01-601-634-3084

The supporting information includes: I. Pricing of cellulose nanomaterials, Spring 2019, II. Atomic Force Microscopy results on as-received CNFs and TOCNFs, surface topology of composites, and surface roughness, III. DSC first and second melting curves from CNF and TOCNF/PVDF composites. IV. Tensile properties of the CNF and TOCNF/PVDF composites.

I. Pricing of cellulose nanomaterials, Spring 2019.

University of Maine Process Development Center.

CNF/CMF	UMaine Cellulose Nanofibrils	Unit Size	Lead Time	Number of Units	Cost per Unit $USD	Cost - $USD S/H will be added
1	Standard Slurry, 3% Solids	1 pound 5-gallon pail	1-2 Days		$50	
2	Standard Slurry, ~15% solids	1 pound 1-gallon pail	2-3 Days		$75	
3	Standard Slurry, Freeze-dried	1/4 pound bag	2-3 Days		$200	
4	High fines slurry, 3% solids	1 pound 5-gallon pail	Made to Order		$75	
5	CNF Slurry – bulk volume	55-gallon barrel	Please Call			
CNC	FPL Cellulose Nanocrystals, acid-hydrolyzed	Unit Size		Number of Units	Cost per Unit $USD	Cost - $USD S/H will be added
6	Slurry, ~11.8% Solids	1 pound 2-gallon pail	1-2 Days		$250	
7	Freeze-dried powder	1/4 pound bag	1-2 Days		$112.50	
8	CNC Slurry – bulk volume	55-gallon barrel	Please Call			
TOCN	FPL Cellulose Nanofibrils, TEMPO-oxidized	Unit Size		Number of 100 gram units	Cost per unit $USD	Cost - $USD S/H will be added
9	Slurry, ~1% Solids	100 grams 4-gallon pail	1-2 Days		$330	
10	Freeze-dried powder	100 grams bag	2-3 Days		$690	
11	TOCN Slurry – bulk volume	55-gallon barrel	Please Call			
					TOTAL for samples	

Figure S1. Nanocellulose pricing (spring 2019), University of Maine Process Development Center.

CelluloseLab, contact@celluloselab.com.

Product Series	Cellulose Lab Catalog Number	Product	Form	Small Package Order		Large Package Order		Extra Large Order
				Size (oven-dry weight basis)	Cost, USD per gram (oven-dry weight)	Size (oven-dry weight basis)	Cost, USD per gram (oven-dry weight)	
NFC series (Cellulose Nanofibrils or nano fibrillated cellulose) or MFC series (micro fibrillated cellulose)	CNF-Slurry	Cellulose Nanofibrils	Slurry, 3.0% solids	1 g – 500 g	$2.00	501 g – 5 kg	$1.75	Please contact us
	CNF-FD	Cellulose Nanofibrils Freeze-dried	Dry	1 g – 200 g	$6.00	201 g – 2 kg	$5.50	
	CNF-CM-Slurry	Carboxymethylated Cellulose Nanofibrils	Slurry, 0.5% - 7% solids	1 g – 60 g	$15.00	61 g – 1 kg	$12.00	
	CNF-CM-FD-P	Carboxymethylated Cellulose Nanofibrils, Freeze-dried, Pulp material	Dry	1 g – 60 g	$15.00	61 g – 1 kg	$12.00	
	CNF-CM-SD-C	Carboxymethylated Cellulose Nanofibrils, Spray-dried, Cotton material	Dry	1 g – 50 g	$20.00	51 g – 1 kg	$15.00	
	CNF-CM-SD-S	Carboxymethylated Cellulose Nanofibrils, Spray-dried, Sisal material	Dry	1 g – 50 g	$25.00	51 g – 1 kg	$20.00	
	CNF-Cationic	Cationic type Cellulose Nanofibrils	Slurry, 0.5% - 7% solids	1 g – 60 g	$20.00	61 g – 1 kg	$17.50	
	CNF-TEMPO-FD	TEMPO (Anionic type) Cellulose Nanofibrils Powder	Dry	1 g – 50 g	$25.00	51 g – 1 kg	$20.00	
	CNF-TEMPO-S	TEMPO (Anionic type) Cellulose Nanofibrils Slurry	Slurry, 0.5% - 7% solids	1 g – 60 g	$20.00	61 g – 1 kg	$17.50	
NCC (or CNC) series (Nanocrystalline Cellulose or Cellulose Nanocrystals)	CNC-Slurry	Cellulose Nanocrystals, acid hydrolysis	Slurry, 11.8% solids	1 g – 500 g	$3.00	501 g – 5 kg	$2.50	
	CNC-FD	Cellulose Nanocrystals Freeze-dried	Dry	1 g – 200 g	$6.00	201 g – 1 kg	$5.50	
	CNC-SD	Cellulose Nanocrystals Spray-dried	Dry	1 g – 200 g	$6.00	201 g – 1 kg	$5.50	
	CNC-CM-SD	Carboxymethylated Cellulose Nanocrystals, Spray-dried, Pulp material	Dry	1 g – 100 g	$10.00	101 g – 1 kg	$8.00	
	CNC-Cationic	Cationic type Cellulose Nanocrystals	Slurry, 1% - 7% solids	1 g – 50 g	$25.00	51 g – 1 kg	$20.00	
	CNC-TEMPO	TEMPO (Anionic type) Cellulose Nanocrystals	Slurry, 1% - 7% solids	1 g – 50 g	$25.00	51 g – 1 kg	$20.00	

Figure S2. Nanocellulose pricing (spring 2019). CelluloseLab.

Nanografi

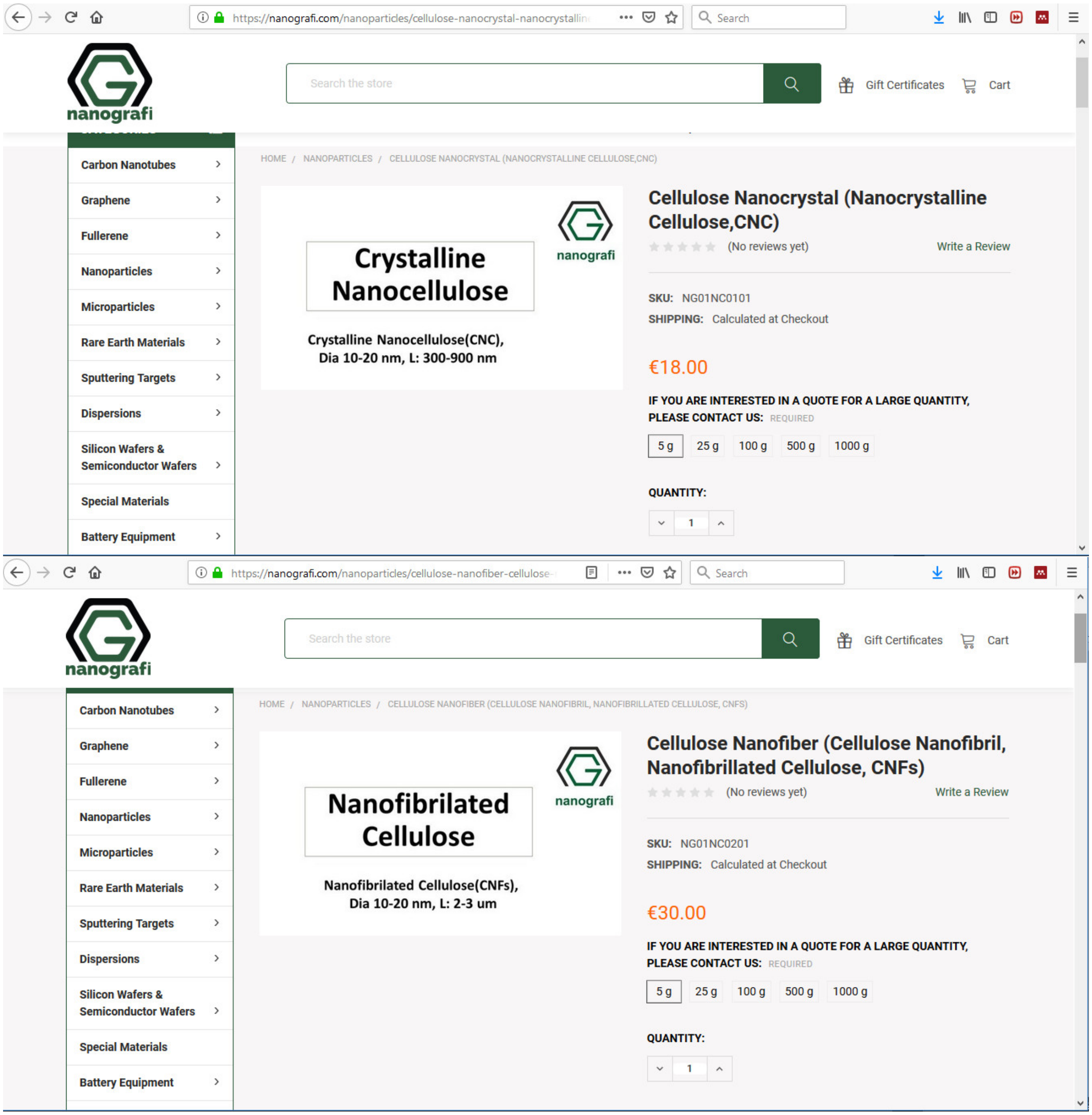

Figure S3. Nanocellulose pricing (spring 2019), Nanographi.

II. Atomic Force Microscopy results

High resolution images of the as-received CNFs and TOCNFs were obtained with Atomic Force Microscopy (Dimension Icon, Bruker) following the preparation method outlined by [30]. Topographical images were captured in the ScanAsyst® mode using a tip with a 2 nm nominal tip radius. AFM data were plotted and processed with Gwyddion [31]. Figure S4 shows 10 µm × 10 µm scans of (a) as-received CNFs and (b) TOCNFs deposited on freshly cleaved mica substrates. Figures S2 and S3 show 10 µm × 10 µm scans obtained from the top (free) and bottom (constrained) surfaces from the PVDF, CNF/PVDF, and TOCNF/PVDF composites. Figures S2 and S3 show the morphology of the PVDF, CNF/PVDF and TOCNF/PVDF top surfaces. Table S1 lists the surface roughness values obtained from the AFM scans shown in Figures S2 and S3.

Table S1. Surface roughness of films obtained from AFM scans.

wt%	Surface Roughness Top surface (rms), nm	Surface Roughness Bottom surface (rms), nm
0	74	2.9
CNF		
0.5	124	3.1
1	188	3.2
2	131	5.1
3	231	7.0
4	293	5.5
5	382	5.3
TOCNF		
0.5	317	4.0
1	255	4.2
2	255	8.0
3	431	6.1
4	327	7.7
5	446	5.3

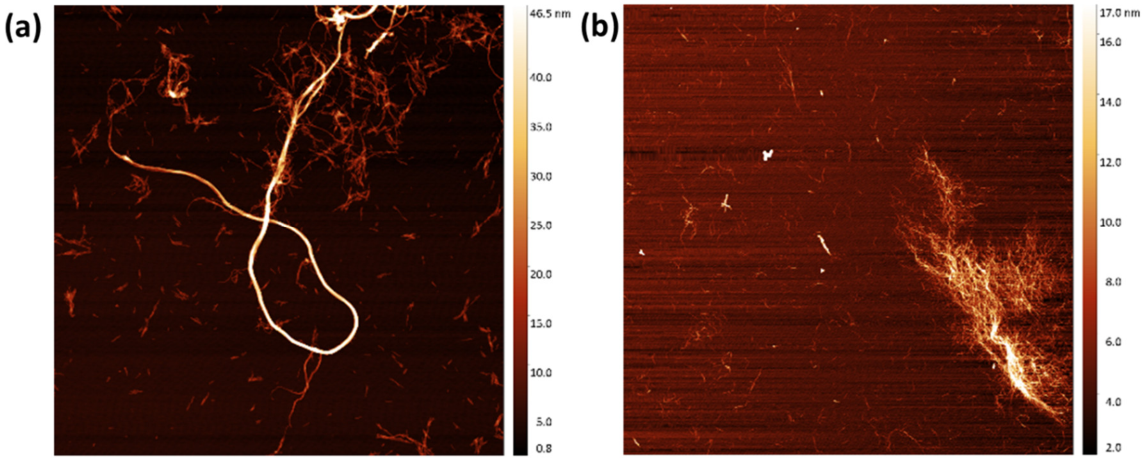

Figure S4. AFM of (a) as-received CNFs, and (b) TOCNFs. Scan size is 10 μm × 10 μm.

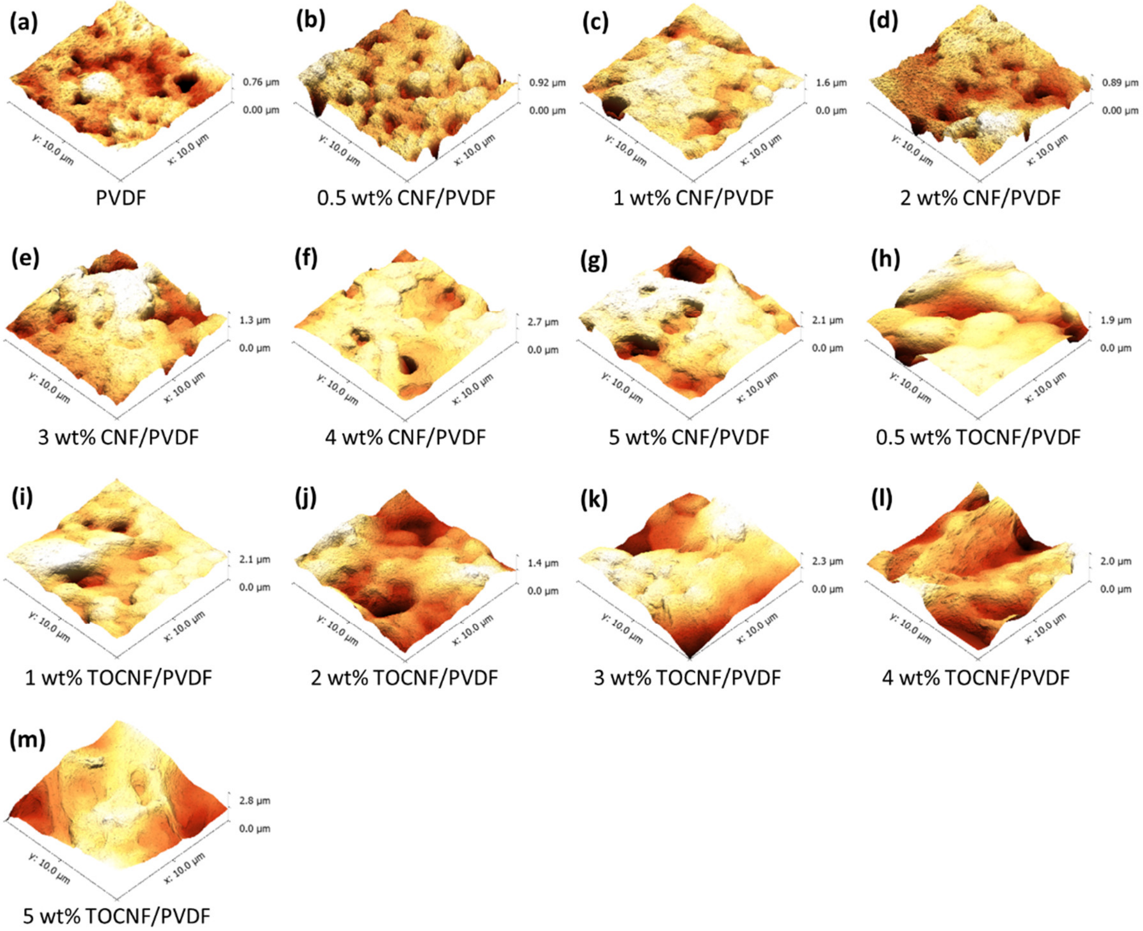

Figure S5. Surface topography of the top (free) surface of (a) PVDF, (b) 0.5,(c) 1,(d) 2,(e) 3,(f) 4,(g) 5 wt% CNF/PVDF, and (h) 0.5, (i) 1,(j) 2,(k) 3,(l) 4,(m) 5 wt% TOCNF/PVDF. Scan sizes are 10 µm × 10 µm.

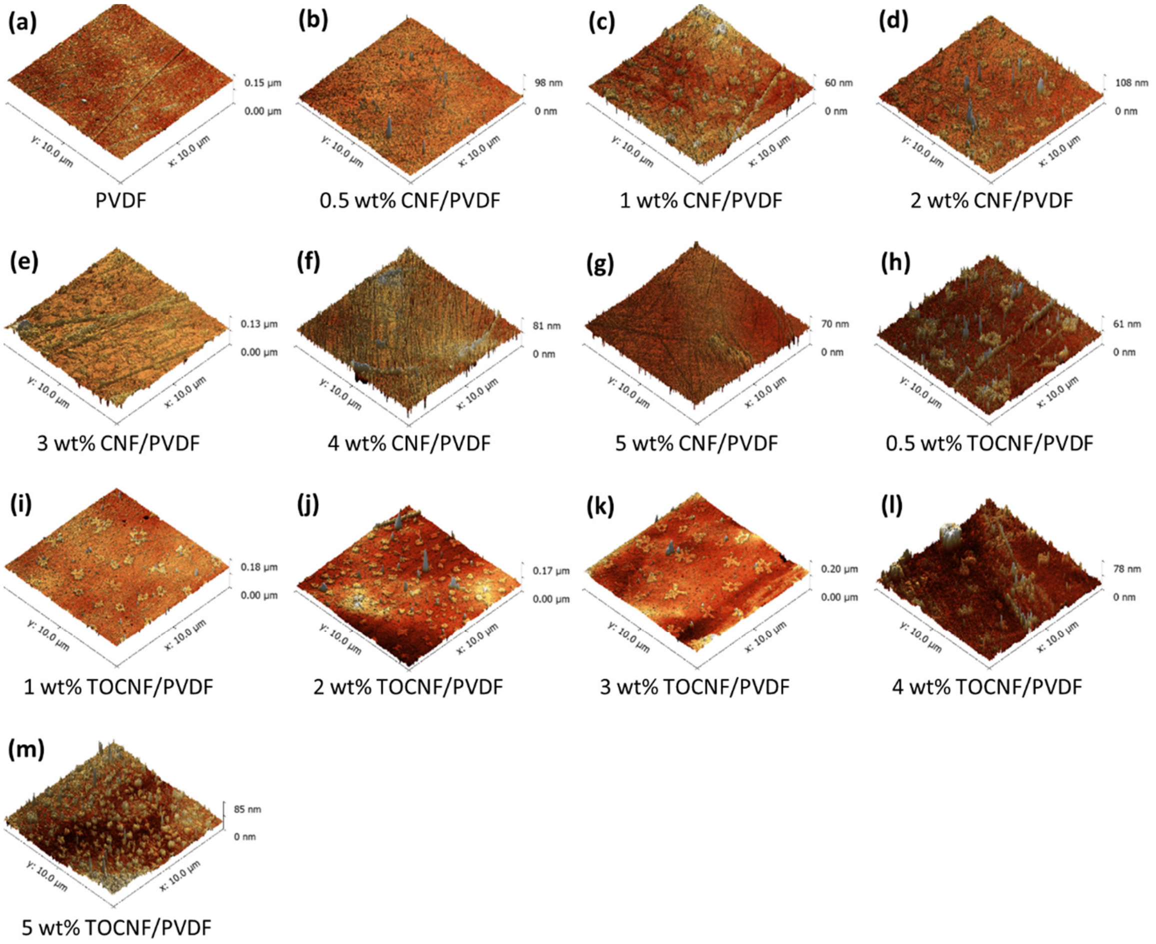

Figure S6. Surface topography of the bottom (constrained) surface of (a) PVDF, (b) 0.5,(c) 1,(d) 2,(e) 3,(f) 4,(g) 5 wt% CNF/PVDF, and (h) 0.5, (i) 1,(j) 2,(k) 3,(l) 4,(m) 5 wt% TOCNF/PVDF. Scan sizes are 10 µm × 10 µm.

Table S2. Summary of Equation 1 fitting results for PVDF, CNF/PVDF, and TOCNF/PVDF films.

	top surface					bottom surface				
**wt%**	θ_i (°)	k ($\times10^{-4}$ s^{-1})	n	r^{2*}	χ^{2**}	θ_i (°)	k ($\times10^{-4}$ s^{-1})	n	r^{2*}	χ^{2**}
0	94.08 ± 0.05	5.6 ± 1.1	0.75 ± 0.04	0.971	0.008	84.87 ± 0.02	1.4 ± 0.3	1.00 ± 0.04	0.979	0.004
CNF										
0.5	91.07 ± 0.02	2.6 ± 0.2	0.95 ± 0.02	0.995	0.002	77.63 ± 0.02	1.8 ± 0.0	1.00 ± 0.02	0.993	0.002
1	93.16 ± 0.02	1.3 ± 0.2	1.00 ± 0.02	0.992	0.002	85.48 ± 0.02	1.8 ± 0.3	1.00 ± 0.03	0.987	0.004
2	105.20 ± 0.03	5.3 ± 0.7	0.70 ± 0.03	0.985	0.003	78.54 ± 0.03	2.4 ± 0.4	0.97 ± 0.04	0.983	0.005
3	-	-	-	-	-	80.43 ± 0.02	2.0 ± 0.2	1.00 ± 0.02	0.994	0.002
4	103.42 ± 0.07	2.1 ± 0.3	0.51 ± 0.03	0.983	0.006	77.70 ± 0.01	1.9 ± 0.2	1.00 ± 0.02	0.995	0.001
5	-	-	-	-	-	78.80 ± 0.03	2.8 ± 0.4	1.00 ± 0.03	0.992	0.005
TOCNF										
0.5	85.68 ± 0.02	1.9 ± 0.3	0.98 ± 0.03	0.989	0.003	81.43 ± 0.01	2.9 ± 0.3	0.89 ± 0.02	0.996	0.001
1	96.10 ± 0.07	8.9 ± 0.2	0.70 ± 0.05	0.960	0.017	77.32 ± 0.01	1.8 ± 0.2	1.00 ± 0.02	0.997	0.001
2	93.20 ± 0.06	14.9 ± 2.8	0.58 ± 0.03	0.973	0.009	79.80 ± 0.01	2.5 ± 0.2	0.91 ± 0.01	0.997	0.001
3	90.43 ± 0.05	7.8 ± 0.2	0.71 ± 0.04	0.970	0.010	75.36 ± 0.01	1.7 ± 0.2	1.00 ± 0.02	0.994	0.001
4	-	-	-	-	-	75.43 ± 0.01	1.6 ± 0.1	1.00 ± 0.02	0.996	0.001
5	-	-	-	-	-	73.23 ± 0.01	1.2 ± 0.20	0.99 ± 0.03	0.984	0.001

*adjusted r^2, **reduced χ^2

III. DSC first and second melting curves from CNF and TOCNF/PVDF composites.

Figure S7 shows the first and second DSC melting curves for the PVDF, CNF/PVDF, and TOCNF/PVDF composites.

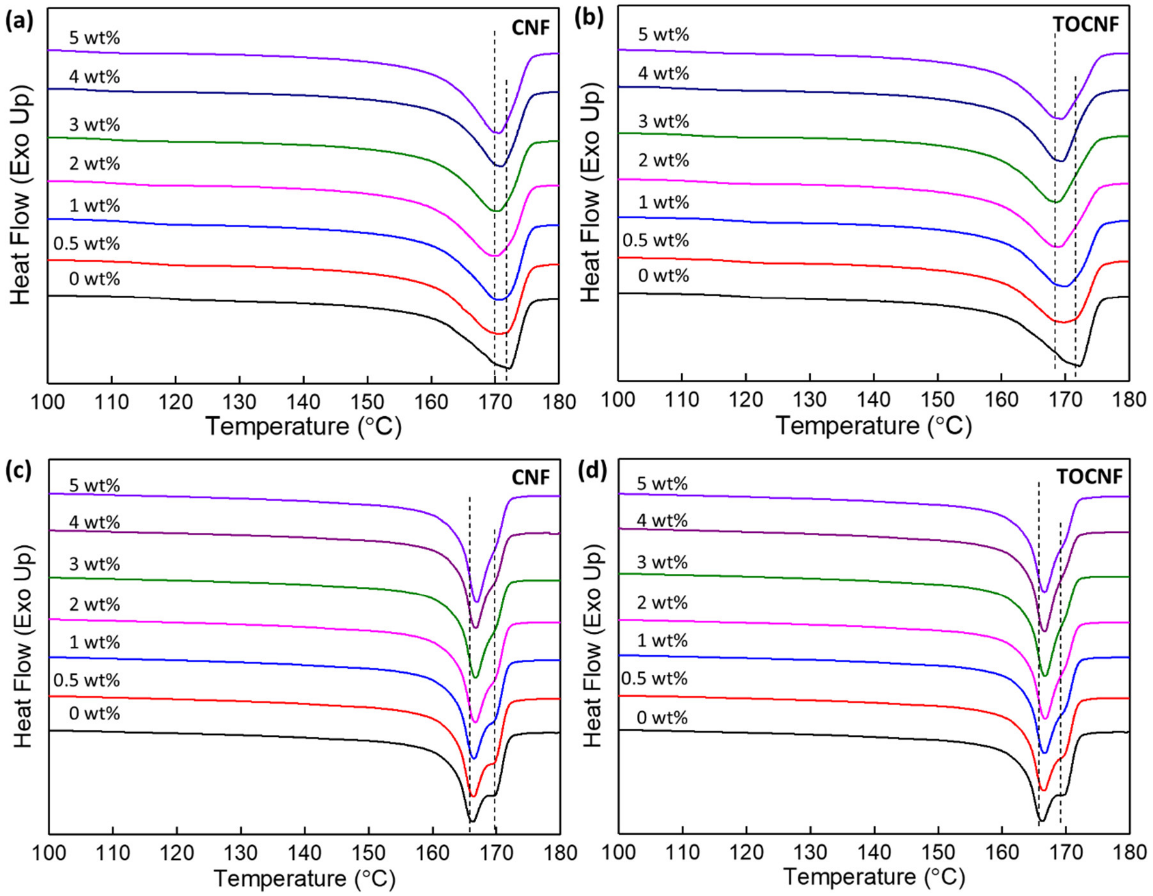

Figure S7. First (a)-(b), and (c)-(d) second DSC melting curves for (a) CNF/PVDF composites and (b) TOCNF/PVDF composites. The results from (b) imply the presence of multiple crystalline phases, melting of imperfect crystalline phase or solid-solid phase transition.

IV. Tensile properties of the CNF and TOCNF/PVDF composites.

The mechanical properties of the composites obtained from the tensile tests (tensile modulus, ultimate tensile strength and % elongation at break) are shown in Figure S8.

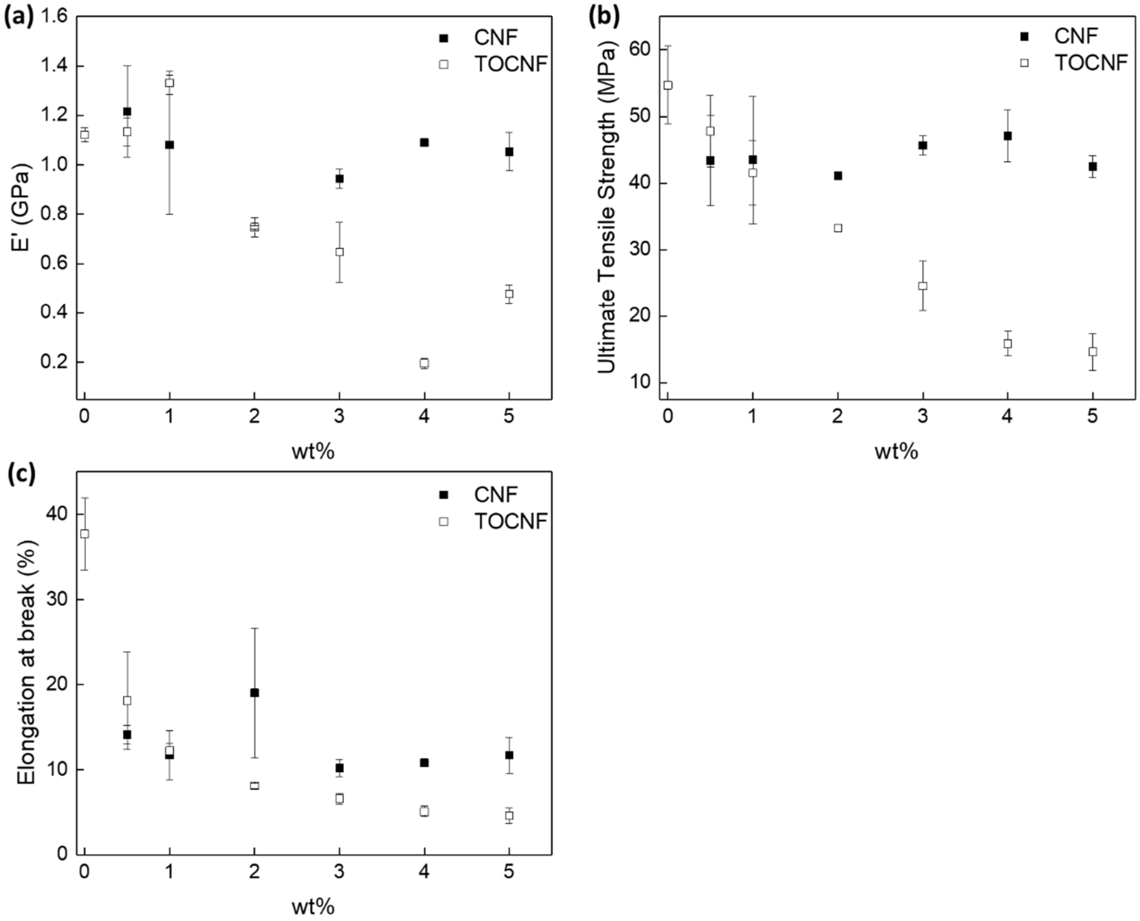

Figure S8. Mechanical properties of the composites as function of CNF/TOCNF wt%, (a) tensile modulus, (b) ultimate tensile strength, and (c) % elongation at break.
